# Results of the enhanced COVID-19 surveillance during UEFA EURO 2020 in Germany

**DOI:** 10.1017/S0950268822000449

**Published:** 2022-03-03

**Authors:** Helena Heese, Adine Marquis, Michaela Diercke, Inessa Markus, Stefanie Böhm, Jasmin Metz, Katharina Katz, Manfred Wildner, Bernhard Liebl

**Affiliations:** 1 Department for Infectious Disease Epidemiology, Robert Koch Institute, Berlin, Germany; 2 Bavarian Health and Food Safety Authority (LGL), Oberschleißheim, Germany

**Keywords:** Enhanced surveillance, public health, COVID-19, infectious disease epidemiology, mass gathering events

## Abstract

In general, mass gatherings might pose a risk to the public health (PH). The UEFA EURO 2020 tournament (EURO 2020) was one of the first mass gathering events since the start of the coronavirus disease 2019 (COVID-19) pandemic in Germany. To allow early detection and response to any EURO 2020-associated impact on the COVID-19-related epidemiological situation, we initiated enhanced surveillance activities using the routine surveillance system in collaboration with the regional PH authority of Bavaria. Several preventive measures regarding the attendance of football matches and public viewing were implemented according to state regulations. We describe the results from the enhanced surveillance during the EURO 2020. In total, five cases who had attended a football match in the stadium of Munich, nine cases, who attended a football match in a stadium outside of Germany, and 123 cases in association with public viewing events were identified by enhanced surveillance. Concluding, the EURO 2020 seems to not have had a major impact on the COVID-19 pandemic development in Germany. Health measures for stadium visitors and the restriction of large public viewing events may have potentially contributed to the low case numbers detected, emphasising the need of appropriate PH surveillance and regulations to limit the potential risk to PH during mass gathering events.

## Introduction

Mass gathering events are prone to become outbreak settings for several communicable diseases and therefore may pose a risk to public health (PH) due to the high number of people participating in the event and the resulting short-term increased likelihood of communicable disease spreading [1–3]. In general, several factors may influence the event-associated risk of disease spreading and outbreaks during an event and include: (a) type of event and expected size, duration and spatial distribution, (b) expected origin/recent travel history of participants, (c) level of infectious disease activity/pressure in the host country at time of the event, (d) amount of public attention and (e) event-related activities during, before and after the event [2, 4, 5]. Especially, large sport events, religious events and cultural festivals might have a short-term impact on local PH [4]. According to the World Health Organization (WHO), outbreaks of various diseases may be likely due to enhanced transmission during a mass gathering event and include meningitis, gastrointestinal and respiratory diseases [6]. Among those, especially respiratory diseases are a major concern of mass gathering events due to the ease of human-to-human transmission and the potential of disease's spread beyond the site of event by returning attendees [7]. Mass gathering events are characterised by an increased density of people coming together from different areas of the world, which is a key prerequisite for providing a suitable context of aerosol transmitted disease outbreaks. Considering the ongoing pandemic situation of coronavirus disease 2019 (COVID-19), mass gathering events may pose an additional threat to PH especially in regards to COVID-19-associated burden of disease within the local population. Therefore, as recommended by WHO appropriate surveillance activities are needed to enable fast detection of deviations from expected disease incidence and allow timely appropriate measures of containment if needed [8].

Due to the COVID-19 pandemic the UEFA EURO 2020 tournament (EURO 2020), originally being scheduled for summer 2020, was postponed to 2021 and took place from 11 June 2021 to 11 July 2021. Staging of football matches was decentralised with venues in 11 different European countries. Germany hosted four football matches in Munich: Germany–France on 15 June 2021, Portugal–Germany on 19 June 2021, Germany–Hungary 23 June 2021 and Belgium–Italy 2 July 2021. All football matches in Germany were held in the Munich Alliance stadium, which is an outdoor stadium. The start of EURO 2020 and timing of football matches hosted by Germany (calendar week (cw) 24–26) followed the end of the third wave of the ongoing COVID-19 pandemic in Germany, which lasted from cw 9 of 2021 to cw 23 of 2021 [9]. At the time of the football matches hosted by Germany (cw 24–26) the nationwide 7-day incidence of COVID-19 was on a continuous downward trend and dropped from 15 (cw 24) to 5 cases/100 000 inhabitants (cw 26) [10]. Measures in place to control COVID-19 during the third wave, which lasted from cw 9 to cw 23 of 2021 [11], such as limited person numbers allowed for private and public meetings or closure of public locations as restaurants and bars, became less strict and pointed towards a cautious opening of daily public life depending on local 7-day incidences.

Due to the federal structure of Germany, the responsibility of defining and implementation of control measures lies within the responsibility of each individual federal state. Within each federal state local PH authorities are in charge of control and response according to the measures in place.

As all football matches hosted by Germany were held in Munich, COVID-19 regulations for the stadium visits of football matches fell under the responsibility of the state of Bavaria. For stadium visits during EURO 2020 football matches in Munich the following regulations were defined: limitation of ticket capacity at 20% of the total seats, individual time-slots for entrance, proof of either complete COVID-19 vaccination, previous COVID-19 infection (‘recovered’, within the 6 months) or a negative polymerase chain reaction (PCR) or antigen test result for severe acute respiratory syndrome coronavirus 2 (SARS-CoV-2) not older than 24 h [12]. Furthermore, general protection measures as keeping physical distance and wearing a FFP2-mask all the time were in place all over Bavaria and had to be applied within the football arena.

Regarding public viewing events at the time of the EURO 2020 control measures for COVID-19 differed between the states. Regulations ranged from only outdoor events to indoor and outdoor events, restrictions to only vaccinated, recovered or negatively tested visitors or any visitors without proof of vaccination, recovery or negative test result.

Due to the nature of the event with expected high numbers of visitors (with international attendance), high public attention and the ongoing COVID-19 pandemic, enhanced surveillance activities, within the routine surveillance for infectious disease were initiated during the time of the EURO 2020.

We aim to describe the results of the enhanced surveillance in order to determine any potential impact of EURO 2020 on COVID-19 infectious disease epidemiology in Germany.

## Methods

**Fig. 1.**
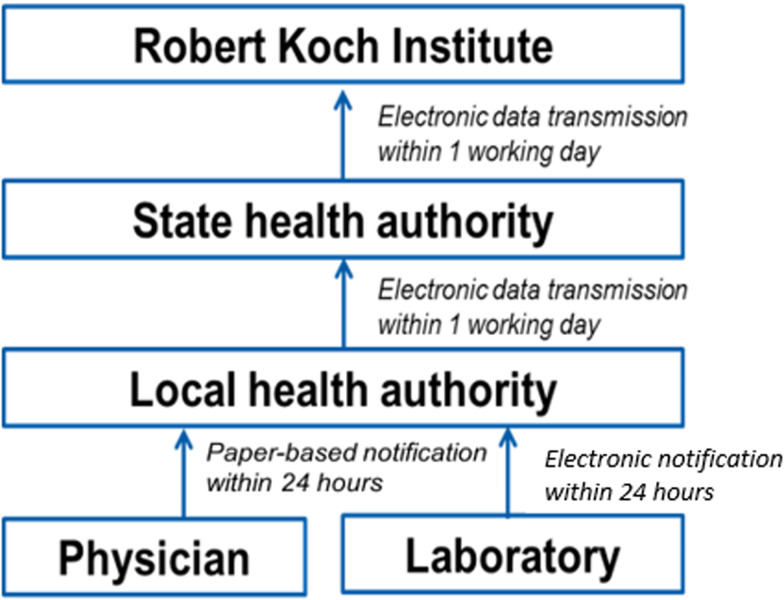
Ways of mandatory notification and transmission within the German surveillance system for infectious diseases.

The German Infection Protection Act defines diseases and pathogens which are mandatory notifiable by physicians and laboratories to the local PH authorities within 24 h of detection. Notified cases are validated according to the specific case definition by the local PH authority, pseudonymised case-based data are transmitted electronically to the state PH office within the next day and from the state PH office to the Robert Koch institute (RKI) as national PH institute within the subsequent day (Fig. 1). At the local level, cases are classified according to the specific case definition. At the time of this report there are 375 local PH authorities reporting to 16 state health authorities in Germany.

At the beginning of 2/2020 COVID-19 infection and the detection of SARS-CoV-2 were added to the list of mandatory notifiable diseases and pathogens [13]. The following information is available on laboratory notification and transmitted from local PH authorities to the state PH authority and RKI: age, gender, date and method of test, detection of a variant of concern (VOC). Besides that, further information needs to be actively investigated by the local PH office and include symptoms, date of symptom onset, hospitalisation status and date, place of exposure, contact to another case and risk factors. This information is also transmitted from local PH authorities to the state PH authority and RKI. This information was used to describe the notified cases.

In agreement with all German states, the enhanced surveillance activities regarding COVID-19 were embedded within routine surveillance activities and to be carried out for a period of the entire EURO 2020 tournament and 2 weeks afterwards (11 June 2021–25 July 2021).

Enhanced surveillance activities included that each case within the observation period that might have had an association with the EURO 2020 was additionally marked with a specific identifier. At a pre-event consultation between national (RKI) and state authorities the nation-wide use of three different identifiers was agreed on to allow the distinction between cases notified in the context of attending a football match within a stadium in Munich, within another stadium in another host city and cases reporting attendance of a public viewing event anywhere. Public viewing event was defined as group gathering in a bar, restaurant or any other public place to watch a EURO 2020 football match broadcast. Furthermore, any cases in the context of privately organised group gatherings to watch a EURO 2020 football match, who were identified by case investigation of the local PH authorities and marked with the defined identifier for public viewing events, were additionally defined as cases in the context of public viewing events in this analysis. Information about the use of identifiers was distributed by the respective state PH offices to their local PH authorities. At RKI, data were regularly screened for any identifier-marked cases. A summary of reported cases was shared with the states. To Bavaria, the state hosting the football matches, a detailed description of cases being marked with the agreed identifiers was reported twice a week.

Cases notified in the context of attending a football match in the Munich stadium were interviewed thoroughly by the responsible local PH authorities in order to identify potential transmission chains and to limit further spreading of disease.

Information about cases notified in the context of attending a public viewing event was not different from information collected by routine surveillance activities. Besides case information received within the national surveillance system, notifications regarding non-German residents were communicated from the local PH authorities to the cross-border contact tracing team at RKI for a further distribution of information to the country of residence.

According to the surveillance case definition only notified COVID-19 cases confirmed by nucleic acid assay or virus isolation were included in the analysis.

We described all COVID-19 cases associated with EURO 2020 by day of notification, place of notification, age, gender, place of exposure, detection of a VOC, symptoms and hospitalisation status. Within the descriptive analysis we distinguished between cases with potential exposure in a stadium of a football match hosted in Munich, Germany, cases with potential exposure in a stadium outside of Germany and cases with potential exposure at a public viewing event in Germany. Only for cases with potential exposure in a stadium of a football match hosted in Munich, Germany, information on the date of the attended football match was collected. In contrast for cases who reported attending a public viewing event, no information regarding the date of the attended public viewing event was available. Local, regional and national case numbers and daily 7-day COVID-19 incidences were derived from routine surveillance data being published daily in reports about the pandemic situation in Germany [10]. Daily 7-day COVID-19 incidences were calculated by dividing the sum of cases reported within the last 7 days by population of the jurisdictional level and multiplying the result by 100 000. Incidences were chosen to compare different geographical areas with different population sizes (the city of Munich, the federal state Bavaria and Germany as a whole).

## Results

**Fig. 2.**
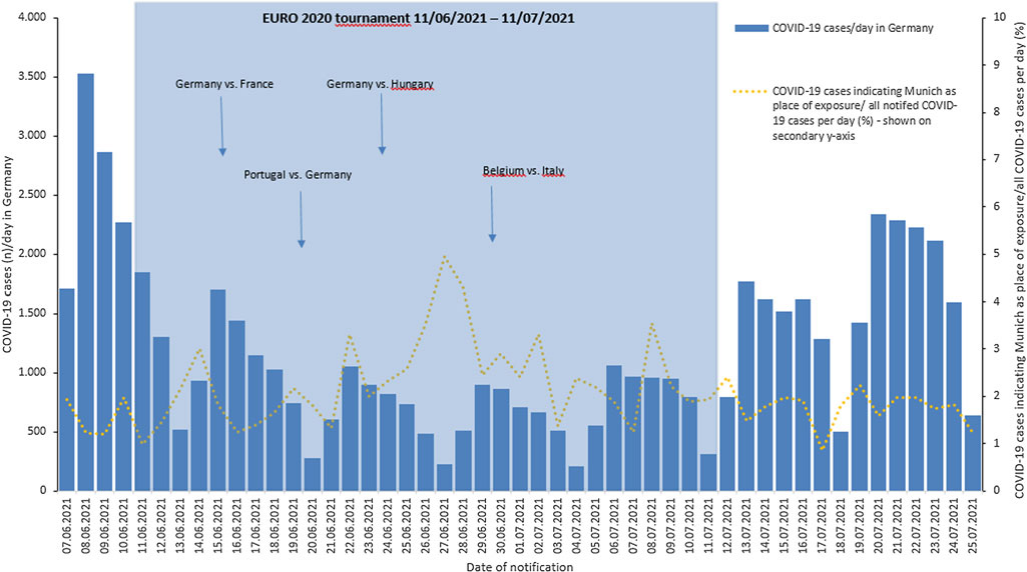
Daily COVID-19 case numbers in Germany and proportion of cases indicating Munich as place of exposure, 7/6/2021–25/7/2021.

Overall, during the time of the enhanced surveillance activities from week 24–26 of 2021, a total of 47 424 COVID-19 cases were reported in Germany. Of those 1088 (1.9%) COVID-19 cases were reported throughout Germany indicating the city of Munich as possible place of exposure. The proportion of cases indicating Munich as a possible place of exposure of all notified COVID-19 cases per day ranged from 0.9% to 5.0% and was highest on 27/06/2021 (Fig. 2). Information about age and gender was available for a total of 47 410 and 47 230 notified cases at the time of enhanced surveillance, respectively. Mean age of all notified cases in Germany was 32 years (median 28, range: 0–99) and 47% were female.

However, only five COVID-19 cases were marked with the specific Munich stadium attendance identifier and were verified to have attended a match in the stadium in Munich during the relevant period. Three of the cases were male, two cases were female and their average age was 48 years (median 48; range: 38–65). All five cases are residents of Bavaria and four of them reside in Munich and one in Landshut.

The first notified case volunteered in the stadium during the match Portugal–Germany in Munich on 19 June 2021. The case developed symptoms on 21 June 2021 and was tested positive for SARS-CoV-2 on 22 June 2021. The case presumably contracted the infection from the person's child, who was a contact person prior to this case's positive test. Overall, the case showed only mild symptoms, had received the first vaccination with BioNTech/Pfizer (Comirnaty) and an antigen test performed before entering the stadium was negative. According to the case's own recollection a mask was worn continuously during the stadium attendance, and no other persons, with whom the case had contact to during the match and knew personally, tested positive afterwards. The person had no increased risk of exposure of infection with regards to their work environment. No information is available on previous stays abroad. The VOC delta was detected.

The second case attended the match Germany–Hungary in Munich on 23 June 2021, and the symptom onset was on 26 June 2021, with only mild symptoms. An antigen test with a positive result was performed on 26 June 2021 and was confirmed by PCR on 27 June 2021. The individual was fully vaccinated with BioNTech/Pfizer (Comirnaty), completing the vaccination series on 28 February 2021, therefore no test prior to stadium attendance was indicated. The case stated that a seat was randomly taken in the stadium. The case returned to Germany from St. Petersburg by plane on 20 June 2021. According to the person's own statement, the only contacts in the stadium were friends and acquaintances. The case travelled to the stadium in a private car and wore a mask continuously during the stay in the stadium. Furthermore, the case was not aware of any other persons, with whom the person had contact to during the match, which tested positive. The person did not report an increased risk of infection with regards to the case's work environment. The VOC delta was detected.

The third case attended the quarterfinal match Belgium–Italy on 2 July 2021 in Munich. The symptom onset was on 5 July 2021, an antigen test with a positive test result was performed on 6 July 2021 and confirmed by PCR on 8 July 2021. The person was unvaccinated and stadium admission was based on a negative antigen test. The person arrived by public transportation and was visiting the stadium with another person, which did not become known as a case. The mask was not worn continuously during the stay in the stadium. The individual developed mild symptoms and was not aware of any other persons, with whom they had contact to during the match, having tested positive. The work environment of the person was not at an increased risk of exposure of infection. The case had no recent stays abroad. The VOC delta was detected.

The fourth and fifth cases also attended the quarterfinal match Belgium–Italy on 2 July 2021 in Munich. The symptom onset for case four was on 5 July 2021, the diagnosis was made on 12 July 2021 and symptom onset for case five was on 8 July 2021 and diagnosis was on 12 July 2021. Admission for both was based on negative antigen tests, as neither individual had received a vaccination. They arrived at the stadium in a private car. The mask was not worn continuously in the stadium, and they stated that they had been surrounded by strangers (<1.5 m). It remained unclear whether the individuals took their assigned seats. Symptoms reported by both individuals were mild, and neither have an occupationally increased risk of infection, nor has there been a previous stay in an area with high COVID-19 incidence. The VOC delta was detected.

No secondary cases with epidemiological link to primary cases who reported visits to a stadium match were identified.

The RKI received 10 notifications of imported COVID-19 cases who reported attendance of a stadium match in other participating countries during the EURO 2020 as of 23 September 2021. The exposure occurred in the Netherlands (one case), Spain (three), Hungary (two), Russia (three) and the exposure of one case is unknown. Eight of the 10 cases were identified as male and two were female. Their average age was 34 years (median 30, range 17–56). Two cases had received one dose of vaccine prior to their infection while all of the remaining cases had not received any dose of vaccine. This results in all 10 cases not being fully vaccinated. For two cases no information on symptoms was available, eight cases developed symptoms while one of them was hospitalised. For seven of the eight symptomatic cases information on the onset of symptoms was available. Their onset of symptoms was between 20 June 2021 and 5 July 2021. However, no information was available about the attended match and respectively the date of the match. The VOC alpha was identified in two of the cases, while delta was identified in five cases. In three cases no information about detection of any VOC was reported.

The cross-border contact tracing team at RKI, which coordinates the communication on COVID-19 cases and contact persons during the COVID-19 pandemic between countries abroad and the state and local health authorities in Germany, received information about two events connected to the EURO 2020.

Two French citizens visiting Munich for the EURO 2020 tested positive with a rapid antigen test on 15 June 2021 in Munich. By the time of notification on 17 June 2021 to RKI they had already returned to France. On 4 July 2021, Italy notified Germany about a COVID-19 case that visited EURO 2020 locations across Europe. In addition to other host cities the case visited Munich from 30 June 2021 to 1 July 2021, developed mild symptoms upon return home and tested positive on 2 July 2021.

Besides the stadium visits in Munich or other participating countries the RKI also received notification of COVID-19 cases who reported attendance at public viewing events of matches during the EURO 2020. As of 23 September 2021, the number of notified cases who reported attendance of a public viewing event amounts to 123. Their average age was 26 years (median 23, range 5–57). Of those 59% (72/123) were identified as male and 41% (51/123) as female. Of all notified cases who reported attending a public viewing event, 60% (74/123) reported symptoms while no case was reported to be hospitalised. Of cases who reported symptoms, information of onset of symptoms was available for 91% (67/74). The onset of symptoms was between 24 June 2021 and 28 July 2021. However, no information was available about the date of the specific attended public viewing event. In 80 (65%) cases the VOC delta was identified, in 22 (18%) cases VOC gamma and in two (2%) cases VOC alpha was reported, while in 19 (15%) no information about the variant was reported. Only 11 (9%) cases were fully vaccinated, eight of them were female. Thirty-five (28%) cases had received one dose of vaccine prior to their infection. The notified cases who reported attending a public viewing event were mainly reported within nine outbreaks (Table 1).

**Table 1.** COVID-19 outbreaks detected in association with a public viewing event during UEFA EURO 2020

Outbreak	Number of cases	Average age	Symptomatic	VOC (WHO nomenclature)
Outbreak 1	73	25 (5–57 years old)	55 (75%)	Delta; unknown
Outbreak 2	26	23 (15–33 years old)	5 (19%)	Gamma; unknown
Outbreak 3	5	18 (17–20 years old)	1 (20%)	Delta; unknown
Outbreak 4	4	31 (20–38 years old)	4 (100%)	Delta; unknown
Outbreak 5	3	48 (40–53 years old)	2 (77%)	Delta; unknown
Outbreak 6	2	35 (29–40 years old)	0 (0%)	Delta
Outbreak 7	2	28 (25–30 years old)	2 (100%)	Unknown
Outbreak 8	2	54 (51–56 years old)	1 (50%)	Delta; unknown
Outbreak 9	2	10 (10–10 years old)	1 (50%)	Alpha

**Fig. 3.**
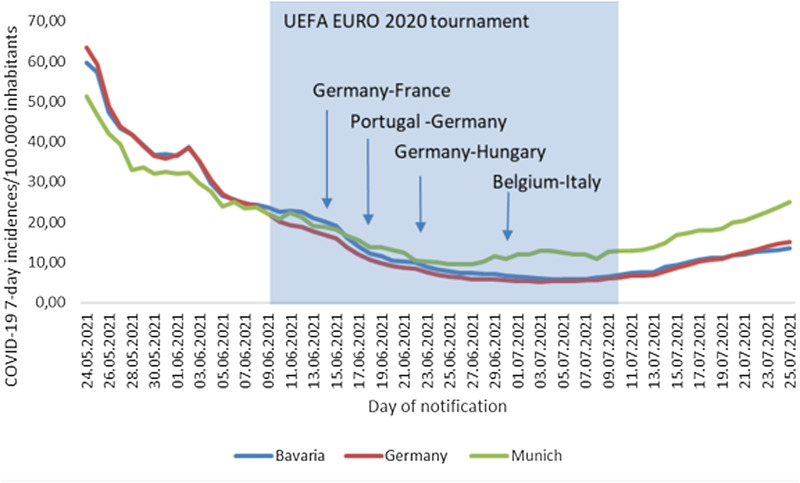
COVID-19 7-day incidence/100 000 inhabitants in Munich, Bavaria, Germany from 24 May 2021 to 25 July 2021.

At the beginning of EURO 2020 on 11 June 2021 the daily 7-day COVID-19 incidences was recorded for Munich, Bavaria and Germany as 22.3, 22.8 and 19.3, respectively. Incidences decreased across all jurisdictional levels until the 26 June 2021 in Munich to 9.5, until the 07 July 2021 in Bavaria to 5.8 and until the 3 July 2021 in Germany to 5.2. From the specific date of the lowest 7-day COVID-19 incidences an increase was recorded across all jurisdictional levels. In Munich, Bavaria and Germany daily 7-day COVID-19 incidences steadily increased to 25.1, 13.6 and 15.0, respectively until the end of the enhanced surveillance activities on 25 July 2021 (Fig. 3).

## Discussion

Since the beginning of the COVID-19 pandemic the EURO 2020 was one of the first mass gathering events being held in Germany at this scale. Due to the nature of the event in general and due to the specific epidemiological situation of the still ongoing COVID-19 pandemic intensified PH alertness was needed, which included the set-up of enhanced surveillance activities. In total, within the German surveillance system for infectious diseases the enhanced surveillance activities identified only 15 notifications of COVID-19 cases who reported attendance of football matches either in Munich or outside Germany and 123 COVID-19 cases that reported attendance at public viewing events. This observation is in contrast to observations made in other countries, where EURO 2020 football matches were also hosted. Scotland reported more than 2000 COVID-19 cases who reported visiting a football match [14]. Similarly, England reported 3071 cases who were potentially infectious and 6784 cases who were infected during EURO 2020 in association with football matches being held in Wembley, London [15]. It is worth mentioning that proof of vaccination, recovery or a negative test result was mandatory in those venues as well as in Munich. However, the official allowed stadium capacity for the specific matches in Wembley stadium (up to 75%) differed to the official allowed capacity in the Munich stadium (20%) [14, 15]. Furthermore, the local COVID-19 epidemiological situation differed between the venues. For example, in Scotland the COVID-19 case numbers were increasing at the time of EURO 2020 which later resulted in the third wave of SARS-CoV-2 [14]. Besides that, it was reported that the stadium attendees were less compliant to infection prevention measures which resulted in stadium areas becoming crowded while ignoring PH measures like face covering and keeping distances at the finale at the Wembley stadium in London [15].

In Germany, local, regional and national daily 7-day incidences of COVID-19 seem to not have been affected strongly by EURO 2020. However, local daily 7-day incidence of Munich despite being overall low, appeared to be slightly higher than regional or national daily 7-day incidences. However, higher daily 7-day COVID-19 incidences in Munich compared to the incidence in the state of Bavaria were already observed before the EURO 2020. The daily proportion of cases indicating Munich as the possible place of exposure increased only slightly by approximately 2 percentage points during the time of EURO 2020. Further factors influencing daily notified case numbers e.g. increasing tourist numbers and travel activities during the time of school summer break which were about to start or have already started at the time of EURO 2020 must also be considered when interpreting the results especially for a touristic destination such as Munich.

Age and gender distribution of cases notified either associated with visits to football matches or public viewing events is in line with anticipated characteristics of persons who might attend EURO 2020, as e.g. young and mid-aged adults and slightly more men. Furthermore, the age distribution of cases might be one reason why only a few cases developed severe symptoms and needed hospitalisation.

Besides enhanced surveillance, specific COVID-19 regulations were in place regarding the attendance of events related to EURO 2020. Compared to other hosting countries, the regulations in Munich appeared stricter, especially in regards to ticket availability, which was limited to 20% of the stadium's total capacity and remained the same for all matches [12]. Furthermore, individual proofs of being either vaccinated, recovered or negatively tested as well as social distancing measures and usage of masks was mandatory at all times. The regulations in place for attendance in the stadium may have potentially limited transmission in the stadium. However, it is worth mentioning that according to media reports there were comprehensive controls before entering the stadium, but during the stay within the stadium some visitors did not adhere to distancing rules and wearing a mask. Additionally, the low level of virus transmission, as evidenced by low population 7-day COVID-19 incidences could have made a higher rate of cases or outbreaks unlikely in Bavaria and Munich.

As expected the number of cases that reported attendance at public viewing events were higher than in relation to stadium visits. Several reasons play into that scenario. Mainly, the number of public viewing events and with that the number of attendees of all occurring events count together were higher than the number of visitors in the stadium during a match since only four matches took place in Munich while an unknown number of public viewing events with an unknown amount of attendees took place during the entire EURO 2020 including a total of 51 matches, which increases the number of potentially exposed and may increase the risk of transmission. Moreover, the regulations for public viewing events were not as strict as in the stadium. Public viewing events took place in less controlled settings like bars, restaurants or at private homes, which offers the possibility of a more intimate setting with private interactions and the possibility of close engagements between strangers without having health measures set in place to decrease the risk of transmission. However, due to the limited information regarding the date of the specific public viewing event which was attended, it remains unclear if the attendance of the public viewing event was the place of exposure.

The impact of EURO 2020 on the slight increase in daily 7-day COVID-19 incidences is unclear. By the start of EURO 2020 some participating states had already started their school summer break while in other states the school summer break was about to begin. Independently from EURO 2020-related travel activities, the school summer breaks may have been associated with an increase of touristic travel activities and higher mobility of the population in general, which may have had an impact on the local 7-day-incidences.

## Limitations

The general limitation of passive surveillance data in terms of completeness and timelines also apply to the enhanced surveillance of COVID-19 cases during the EURO 2020. Enhanced surveillance during EURO 2020 was mainly based on surveillance activities integrated in the routine system and no active case finding was conducted. Furthermore, cases might have been detected by the local health authorities but not been marked of being associated with EURO 2020 events. For example, for the period of EURO 2020 attendance of a sport event as possible place of infection was reported for additional 29 notified COVID-19 cases. However, further information about the attended sport event was not given making it difficult to decide a possible association to EURO 2020. Therefore, it is possible that cases that attended relevant EURO 2020 events have been missed.

Additionally, the quality of the outbreak data is not sufficient to assess whether the reported outbreak refers to a common visit to a public viewing event as the place of infection or whether it is a matter of transmission chains following the event. As the size of the events is also not known, event-specific attack rates could not be calculated. Furthermore, information regarding the overall settings and specific dates of the public viewing events is missing in the surveillance data. Therefore, it was not possible to differentiate between indoor and outdoor public viewing events. It is worth mentioning that indoor public viewing events would be prone to higher transmission rates [16, 17]. Another limitation is that the onset of symptoms of the cases who reported attending a public viewing event was available in the surveillance data but no information on the date of the specific attended public viewing event was reported. Therefore, it is not clear whether the case acquired the infection at the public viewing event or at another occasion. However, in contrast to that the information on which stadium match in Munich was visited by the cases who reported stadium attendance, is available in the surveillance data. It needs to be considered that for all cases who attended a football match in Munich the time between the football match and symptom onset was between 2 and 3 days and thereby below the medium incubation period of 5.8 days [18]. Surveillance data cannot prove indisputably whether attending a EURO 2020 match was the place of infection or if the infection occurred in another setting, e.g. private gathering, public transportation, etc. Furthermore, with the available data it is not possible to describe the exact root of the incidence fluctuations. False-negative antigen test may have contributed to disease spread within the stadium. Active case finding during mass events, including follow-up testing of cases after their participation in a mass event, might be another option for a more detailed insight into the PH impact of a specific event. However, given the high workload caused by the COVID-19 pandemic a cost–benefit assessment would support the decision making on the added value of active surveillance.

## Conclusion

In accordance with the reported cases and the trend in incidences, it seems that the EURO 2020 did not have a major impact on the pandemic development in Germany. It seems reasonable to assume that this observation is at least partially attributed to the strict PH measures for stadium visitors and the restriction of large public viewing events. In conclusion, an implementation of PH measures besides high vaccination coverage may help to limit transmission during mass gathering events even in low incidence phases.

## Data Availability

The data that support the findings of this study are openly available at https://github.com/robert-koch-institut/SARS-CoV-2_Infektionen_in_Deutschland with doi: 10.5281/zenodo.4681153.
